# Anxiolytic Effect of Alcohol-Water Extracted Suanzaoren-Wuweizi Herb-Pair by Regulating ECS-BDNF-ERK Signaling Pathway Expression in Acute Restraint Stress Male Rats

**DOI:** 10.1155/2020/2078932

**Published:** 2020-06-17

**Authors:** Cheng-Bo-Wen Zhao, Jie Liu, Jin-Li Shi, Shao-Nan Wang, Yong-Sheng Ding, Shuai He, Ming-Xuan Lin, Jing Luo, Yi-Nan Jiang, Li-Hua Bian, Zi-Wei Yao, Qiu-Yu Li, Xiao-Mei Wang, Jian-You Guo

**Affiliations:** ^1^School of Chinese Materia Medica, Beijing University of Chinese Medicine, Yangguang South Street, Fangshan District, Beijing 102488, China; ^2^Capital Institute of Pediatrics, No. 2 Yabao Road, Chaoyang District, Beijing, China; ^3^CAS Key Laboratory of Mental Health, Institute of Psychology, Chinese Academy of Sciences, 4A Datun Road, Chaoyang District, Beijing 100101, China

## Abstract

Herb-pairs are the basic units of composition in Chinese herbal formulae, where the bridge linking Chinese medicine and prescription consists of two Chinese medicine herbs. The Suanzaoren-Wuweizi herb-pair (SWHP) is commonly used as a sedative or tranquilizer. SWHP has been demonstrated to exert an antianxiety effect in animal models of anxiety. However, little information about its mechanism is available and the effects of SWHP have not been investigated. This study examined the effects of SWHP on ameliorating anxiety-like behaviors by regulating endocannabinoids system (ECS)—brain-derived neurotrophic factor (BDNF)—extracellular regulated protein kinases (ERK) signaling pathway expression, induced by restraint stress (RS) procedures. The antianxiety effects of SWHP on RS rats were then examined through the open-field test (OF) and the elevated plus maze test (EPM). The concentration of BNDF, ERK1/2, p-ERK1/2, cAMP-response element binding protein (CREB), and p-CREB expression in the prefrontal cortex and hippocampus of the rats was then measured by western blot. The number of positive cells of CB1 and CB2 in the rats' hippocampus CA1 region was measured by immunohistochemistry. These results gave compelling evidence that SWHP could modify anxiety-like behaviors of RS rats through regulation of the ECS-BDNF-ERK signaling pathway. Our study demonstrated that SWHP improved anxiety-like behaviors in RS rat models by regulating the ECS-BDNF-ERK signaling pathway. The findings indicate that SWHP may have a therapeutic application in the RS model of anxiety disorder, which proposes a potential new direction for research into anxiety disorders regarding mechanisms and the development of novel antianxiety drugs.

## 1. Introduction

Anxiety disorder is a nervous disorder characterized by anxiety, tension, panic, autonomic nervous disorder, muscle tension, and other symptoms. It may be present when a person has feelings of nervousness or fears that interfere with his/her social, school, or work life [1]. As we know, anxiety disorders are among the most common psychiatric disorders that affect all age groups in the general population, where the incidence rate continues to increase year by year. Anxiety disorders are also among the costliest mental disorders, regarding both morbidity and economic costs [2, 3]. Antianxiety drugs such as diazepam, benzodiazepines, and selective serotonin reuptake inhibitors (SSRIs) (amongst others) exhibit different degrees of side effects when undergoing treatment. Benzodiazepines have been used in the treatment of several forms of anxiety, but these compounds include side effects such as amnesia, muscle relaxation, sedation, and potential dependence [4–6]. Regarding SSRIs, the MHRA and FDA warned that SSRIs not only increased the risk of suicidal behavior and self-harm, but also were of minimal benefit when it came to the treatment of children and adolescents [7, 8]. Side effects such as these therefore limit their use in clinics. The development of anxiety-reducing (anxiolytic) drugs has been a major focus in both pharmaceutical industries and academic neuropsychiatric research [9, 10]. Therefore, the development of anxiolytic drugs without adverse effects is extremely urgent for the treatment of anxiety disorders.

Traditional Chinese Medicine (TCM) has formed a series of therapies for the treatment of anxiety disorders, and it plays an important role in the field of mental diseases with some advantages over western medicine. Compared with western medicine, TCM is safer and more effective in treating anxiety, with fewer side effects. With the increasing incidence of anxiety disorders, many patients have chosen TCM or integrated TCM and western medicine for treatment [11, 12]. Herb-pairs, the basic units of composition in Chinese herbal formulae, usually consist of two Chinese medicine herbs [13]. Suanzaoren is the first choice for sedatives and tranquilizers [14] and is the mature seed from *Ziziphus jujube*. From our previous research, we found that spinosin of Suanzaoren exerts anxiolytic-like effects [15]. Wuweizi, the fruit of *Schisandra chinensis*, is a common tranquilizer [16], which can be combined with a variety of other Chinese medicine herbs. Studies found that Suanzaoren and Wuweizi are usually combined with a variety of Chinese medicine herbs in clinics for use as sedatives and tranquilizers [17–20]. Some studies suggested that schisandrin could reverse stress-induced anxiety levels, showing antianxiety effects in mouse models [21, 22]. SWHP, as sedatives and tranquilizers, are commonly used in clinical practice and often appear in TCM prescriptions as well as modem Chinese medicine compounds.

The endocannabinoids system (ECS) is a type of lipid signal system, which is composed of endocannabinoids, cannabinoid receptors, endocannabinoids synthetase, degradation enzymes, and transporters. These are present in the central nervous system (CNS) and peripheral tissue and have extensive physiological functions. Studies have found that cannabinoid substances bind to cannabinoid receptors to activate signaling pathways in multiple cells, including MAPK/ERK pathways. These MAPK/ERK pathways regulate a variety of cellular processes in both nature and developing tissues [23]. Brain-derived neurotrophic factor (BDNF) is the most abundant neurotrophic factor in the CNS, and its cellular mechanism is related to neuroplasticity. There are two primary signaling pathways of BDNF, the mitogen-activated protein kinase (MAPK) signal pathway and phosphoinositide 3-kinase signal pathway (PI-3-K). Most of the time, the MAPK signaling pathway is playing the most important role. Studies have found that BDNP plays a role not only in the treatment of depression but also in pervasive anxiety disorder [24]. One study showed that physical and emotional stressors could induce the upregulation of ERK1/2 phosphorylation in central neurons (a member of the MAPK family). Thus, the ERK1/2 molecule was found to be key in the stress-induced intracellular signal transduction pathways of brain circuitry neurons and could be used as an endogenous morphological marker of neuronal activity in the brain [25]. Stress could lead to abnormalities in neural circuits related to cognition, decision-making, anxiety, and depression [26]. The prefrontal cortex and hippocampus are associated with mood-related symptoms of stress-related disorders [27] and their neuronal populations to altered behavioral responses to acute stress. As we knew, the prefrontal cortex and hippocampus are anatomically connected, and brain activity within them was relatively closely synchronized. The prefrontal cortex and hippocampus play an important role in anxiety, and they are cooperating during anxiety [28], being mediated by the corresponding receptors on the neuron membrane, resulting in the onset of numerous essential signaling pathways, like the BDNF signal pathway.

Therefore, in this experiment, we hypothesized that alcohol-water extracted SWHP may have an anxiolytic-like effect in restraining stress-induced animal models of anxiety disorders. We investigated its possible mechanism of action regarding the regulation of ECS-BDNF-ERK signaling pathway expressions.

## 2. Materials and Methods

### 2.1. Drugs and Dosages

Suanzaoren (*Ziziphus jujuba* var. *spinosa* (Bunge) Hu ex H.F.Chow, No. 20160122) and Wuweizi (*Schisandra chinensis* (Turcz.) Baill, No. 20150601) were authenticated by Professor Jinli Shi (School of Chinese Materia Medica, Beijing University of Chinese Medicine, Beijing, China) according to the *Chinese Pharmacopoeia* [29]. Voucher specimens were deposited at Institute of Chinese Materia Medica, Beijing University of Chinese Medicine, Beijing, China. Diazepam was obtained from Yimin Pharmaceutical Factory (Beijing, China, SFDA Approval No. H11020898). Sodium Chloride Injection, 500 mL/bottle, was purchased from Shijiazhuang Fourth Pharmaceutical Co., Ltd. (SFDA Approval No. H13023200). The drug dosages in the experiment included SWHP (0.75 g/kg/d), SWHP (1.5 g/kg/d), SWHP (3.0 g/kg/d), and diazepam (1.0 mg/kg/d). The drug SWHP was extracted by alcohol-water, and the compatibility proportion was 2 : 1.

The Suanzaoren (10 g) and Wuweizi (5 g) plant materials were placed in a 500 mL round-bottom flask. The plant materials soaked for 30 minutes with 8 times 80% alcohol and then boiled by heat under reflux for 1 h. The decoction was filtered through filter paper. The residue was boiled with 10 times water for 1 h and then filtered and mixed with the first-stage extract solution. Finally, the combined extract solution was taken to dryness and then stored at sealed and drying container.

### 2.2. Alcohol-Water Extract Analysis by UPLC-LTQ Orbitrap MS

We established the stable condition of UPLC-LTQ Orbitrap MS to analyze the components of SWHP under the positive/negative ion mode, which had a good separation degree and comprehensive cracking information. Chromatographic analysis was performed using a Thermo Scientific Dionex UltiMate 3000UHPLC Plus Focused (Thermo Scientific, USA) equipped with a binary pump, an autosampler, a column oven, and a Diode-array Detector (DAD). Samples were separated on an Agilent ZORBAX SB-C18 column (4.6 mm × 250 mm, 5 *μ*m). The mobile phases consisted of solvent acetonitrile (A) and water containing 0.1% formic acid (B) with a liner gradient program: 0–5 min, 2%–3% A; 5–8 min, 3%–9% A; 8–30 min, 9%–11% A; 30–32 min, 11%–18% A; 32–52 min, 18%–19% A; 52–67 min, 19%–25% A; 67–74 min, 25%–43% A; 74–78 min, 43%–52% A; 78–79 min, 52%–55% A; 89–114 min, 55%–95% A. The flow rate was set at 0.4 mL/min, and column temperature was 30°C. The sample solution (4 *μ*l) was injected into the HPLC system.

We used the LTQ-Orbitrap XL mass spectrometer (Thermo Scientific, USA) to MS analysis. The mass spectrometer was connected with heated electrospray ionization (HESI) source and operated in both positive and negative ion modes. The mass scan was from 100 to 1500 *m*/*z*. The source voltage was 4.0 kV; the drying gas flow rate was 15 L/min; the drying gas temperature was 350°C; the capillary temperature was 250–350°C; the collision voltage was 6–10 V. The components were identified according to the secondary quality spectrum fragments and the related studies reported by Suanzaoren and Wuweizi. A total of 30 compounds were identified including Gomisins E, G, J, K1, K2, M1, M2, L2, Zizyphusine, Ceanothic acid, and Lignans (Table 1).

### 2.3. Animals

A total of 60 male SD rats (10 rats per group), weighing 150–170 g, were obtained from the Chinese Academy of Military Medical Sciences. Animals were individually caged and were maintained in a standardized environmental condition: 22 ± 2°C, about 46% humidity and on a 12 h/12 h light/dark cycle (light on 7 : 00–19 : 00). The rats were fed with water and food available *ad libitum* before experiments. The Animal Care and Use Committee of the Institute of Psychology of the Chinese Academy of Sciences and the National Institutes of Health Guide approved the experimental procedures for Care and Use of Laboratory Animals. All of the experiments were performed in a quiet room under dim red light between 8 : 00 AM and 12 : 00 PM. All efforts were made to minimize the number of animals used and their suffering [15].

### 2.4. Restraint Stress

As previously described, restraint stress (RS) was used to induce anxiety-like behaviors in rats [30]. Animals were placed in restraining tubes in which they were not physically compressed or experiencing pain [31] and did not inhibit their breathing. After restraint, the rats were returned to their home cages and given food and water *ad libitum*. There was no restriction on the rats in the control group. Restraint was repeated, consisting of 30 minutes of restraint on each of 3 consecutive days, and then the behavioral tests were conducted after the third day.

### 2.5. Procedure

**Figure 1:**
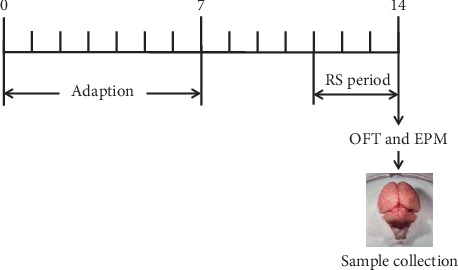
The flow chart of animal experiments.

The rats were adapted for 1 week before the experiments. The rats of the restraint stress model experiment were given drugs orally once a day for 7 days. All drugs mentioned were dissolved in normal saline and prepared before use. The control group and restraint stress group (RS group) were given normal saline as placebo. The volume of administration was 1 mL/100 g of body weight. On the 12^th^–14^th^ day, except for the control group, the others were given restraint stress modeling. Behavioral tests were conducted 1 h after the last administration on the 14^th^ day. Immediately after all behavioral tests, brain samples were taken (Figure 1).

### 2.6. Elevated Plus Maze Test (EPM)

Anxiolytic activity was measured using the elevated plus maze (EPM) test [32]. This model has been widely validated for measuring anxiolytic-like effects in rodents [33]. The device consisted of two opposite open arms (50.8 cm × 10.2 cm) and two closed arms (50.8 cm × 10.2 cm × 40.6 cm) in a cross configuration. The arms were connected to a 10.2 cm × 10.2 cm central platform, and the device was elevated 50 cm above the floor. The test was conducted under quiet and dim light conditions [34]. Before the test, animals were individually placed in the 45 cm × 30 cm × 15 cm plastic boxes for 5 minutes of free exploration. The animals were then placed in the center of the maze facing an individual open arm. The test was maintained for 5 minutes. Both of the number of entries into and the time spent on each of the closed arms, open arms, and central platform were measured with infrared technology. The percentage of entries to the open arms ((open arm entries/total arm entries) × 100) and the percentage of time spent in the open arms ((open arm time/total arm time) × 100) were calculated for the index of antianxiety effect.

### 2.7. Open-Field Test (OF)

The open-field device consisted of an open-field chamber (radius 90 cm × high 60 cm). 5 minutes before the test, animals were individually placed in 45 cm × 30 cm × 15 cm plastic boxes for free exploration. Then explored animals were then individually placed on the edge of the open field. The test was initiated by placing a single rat in the open field and allowing it to move freely for 5 minutes [35]. A digital camera placed above the open field with an automatic video tracking system recorded their activities. The number of entries into and the time spent on the central area (45 cm radius) were measured over the 5-minute observation period. A change in counts was considered an alteration in the locomotor activity of the experimental subject caused by the drugs [36].

### 2.8. Immunohistochemistry

Rats were anesthetized with 10% pelltobarbitalum natricum and were sacrificed by decapitation after the completion of behavioral tests immediately (*n* = 8 per group). The hippocampus and prefrontal cortex were dissected and fixed in a 10% neutral buffered formalin solution for 48 h. The brain sections (5-*μ*m) were dehydrated and embedded by paraffin for conventional immunohistochemical treatment.

Paraffin sections were conventionally dewaxed with water, and the sections were incubated with 3% H_2_O_2_ for 10 minutes at room temperature. They were then rinsed by phosphate buffered solution (PBS) for 3 minutes, three times. Sections were then put into citrate buffer salt and bought to high temperatures using thermal remediation. Once rinsed again with PBS, sections were blocked with 5% normal serum for 20 minutes at room temperature. Sections were then incubated with the anti-Cannabinoid Receptor I antibody (1 : 1000, ab23703, ABCAM, UK) or anti-Cannabinoid Receptor II antibody (1 : 20, ab3561, ABCAM, UK) at 4°C overnight. After incubation with biotinylated anti-rabbit IgG antibody (1 : 100) for 15 minutes at 37°C, sections were incubated with horseradish enzyme labeling Streptomyces albumen working fluid (1 : 100) for 15 minutes at 37°C. The immunostaining reaction was developed using the DAB (3, 3′-diaminobenzidine) peroxidase substrate kit and then restained with haematoxylin. Finally, sections were mounted on glass slides after dehydration. Image acquisition and processing are carried out by means of microscope, computer, and image analysis software. The positive cells of CB1 and CB2 in the hippocampal CA1 region of the rats were, respectively, conducted under the microscope of 40 × 10 times. Two discontinuous slices were taken from each rat, and three nonoverlapping views were randomly chosen from each specimen to be imaged for counting. The total number of positive cells in the three visual fields represented the number of positive cells in the CA1 region of the hippocampal region of the rat and was statistically treated.

### 2.9. Western Blot

The hippocampus and prefrontal cortex were dissected and stored at −80°C until analysis after the behavioral tests and sacrifice (*n* = 8 per group). All tissues (200 mg) were homogenized in RIPA lysis buffer and sonicated for 4 seconds, 3 times in an ice bath. They were then centrifuged at 14,000 rpm for 20 minutes at 4°C. The supernatant was taken to assess p-ERK1/2, ERK1/2, p-CREB, and CREB, as well as the internal reference protein *β*-action expression. After glue pouring, sample loading, and electrophoresis, the transmembrane operation was carried out and conducted with 0.23 A constant current for 1 h. The membranes were incubated with the primary antibodies, i.e., the Phospho-p44/42MAPK (ERK1/2) (1 : 1000, CTS, USA), p44/42MAPK (ERK1/2) (1 : 1000, CTS, USA), Phospho-CREB (1 : 1000, CTS, USA), CREB (1 : 1000, CTS, USA), and *β*-action (1 : 1000, CST, USA) over night at 4°C. The membranes were then incubated with the secondary antibodies, i.e., goat anti-rabbit HRP (1 : 10,000, EarthOx, USA) for 1 h. Band signal was visualized by the electrochemiluminescence (ECL) kit and was detected by a fluorescence scanner. The quantification of specific bands was analyzed by software (FluorChem E, Protein Simple, USA), and data was acquired, calibrated, and analyzed.

### 2.10. Data Analysis

Data was expressed as mean ± standard error of the mean (SEM). The statistical analysis was performed using one-way analysis of variance (ANOVA), followed by the Student–Newman–Keuls *post hoc* test. In cases of significant variation, the individual values were compared using Dunnett's test. Values of *p* < 0.05 were considered statistically significant. GraphPad Prism 5.0 (GraphPad Software Inc., La Jolla, CA, USA) software and SPSS 17.0 (SPSS Inc., Chicago, IL, USA) were employed.

## 3. Results

### 3.1. Antianxiety Effect of  SWHP in OF

**Figure 2:**
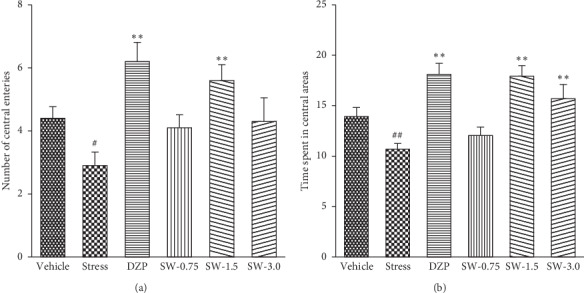
Effects of SWHP on the number of central entries, time spent in central areas, and the total distance in the OF in restraint stress rats. The open-field test was measured for 5 min. (a) Number of central entries. (b) Time spent in central areas. Data represent mean ± SEM. ^#^*p* < 0.05 or ^##^*p* < 0.01 vs. control group and ^*∗*^*p* < 0.05 or ^*∗∗*^*p* < 0.01 vs. RS model group. One-way ANOVA with Student–Newman–Keuls *post hoc* test.

As shown in Figure 2, significant differences by one-way ANOVA were found in the number of central entries [*F* (5, 54) = 4.907, *p* < 0.05] and time spent in the central area [*F* (5, 54) = 9.273, *p* < 0.01] among groups. Diazepam (1.0 mg/kg) significantly increased the number of central entries and the time spent of central area compared with the model group (*p* < 0.01, *p* < 0.01). SWHP at a dose of 1.5 g/kg significantly increased the time spent and the number of central entries (*p* < 0.01, *p* < 0.01). SWHP at a dose of 3.0 g/kg significantly increased the time spent of central area (*p* < 0.01).

### 3.2. Antianxiety Effect of SWHP in EPM

**Figure 3:**
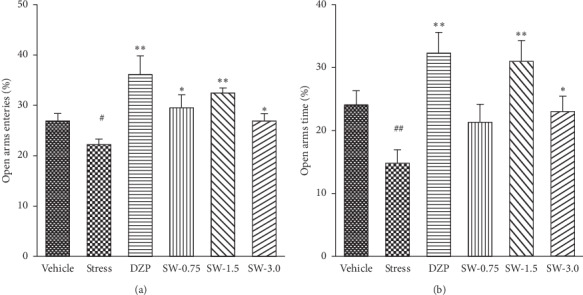
Effects of SWHP on the percentage of entries in open arms and the percentage of time spent in open arms in restraint stress rats. Time spent in the EPM was measured for 5 minutes. (a) The percentage of entries in open arms. (b) The percentage of time spent in open arms. Data represents mean ± SEM. ^#^*p* < 0.05 or ^##^*p* < 0.01 vs. control group and ^*∗*^*p* < 0.05 or ^*∗∗*^*p* < 0.01 vs. RS model group. One-way ANOVA with Student–Newman–Keuls *post hoc* test.

As shown in Figure 3, there were significant differences by one-way ANOVA in the percentage of open arm entries (*F* (5, 54) = 5.172, *p* < 0.05) and the percentage of time spent on the open arms (*F* (5, 54) = 5.485, *p* < 0.01) among groups. Diazepam (1.0 mg/kg) significantly increased the percentage of entries in open arms and time spent in open arms compared with the control group (*p* < 0.01, *p* < 0.01). SWHP significantly increased the percentage of entries in open arms (*p* < 0.05, *p* < 0.01, *p* < 0.01). SWHP at doses of 1.5 g/kg and 3.0 g/kg significantly increased the percentage of time spent in open arms (*p* < 0.01, *p* < 0.05).

### 3.3. Effects of SWHP on ECS Signaling Pathway Expression in RS Rats

**Figure 4:**
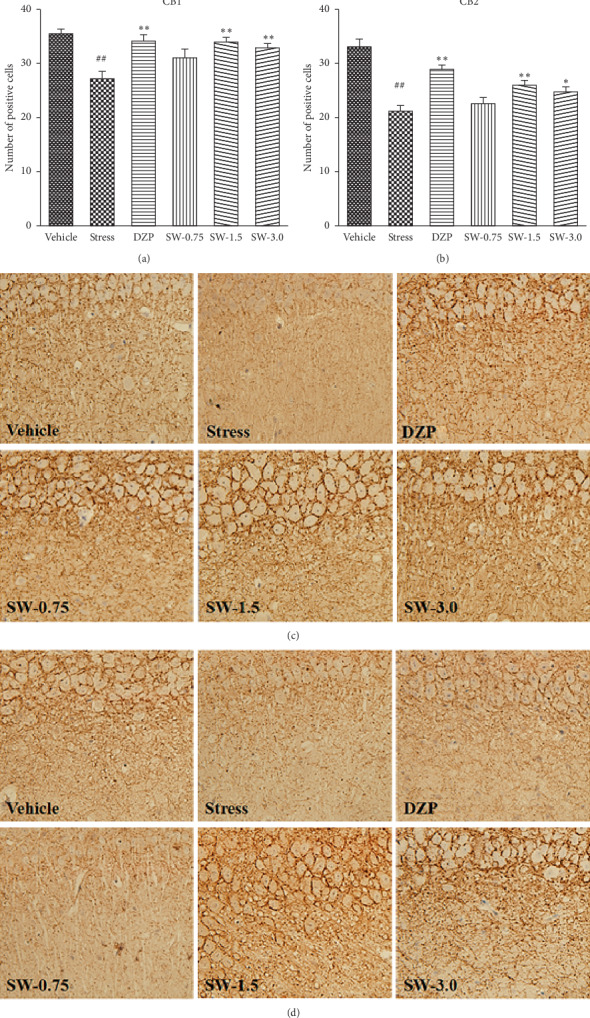
Effects of SWHP on the ECS expression of CB1 and CB2 in the hippocampal CA1 region in restraint stress rats. The positive cells were stained in CB1 and CB2 in the hippocampal CA1 region. Data represent mean ± SEM. ^#^*p* < 0.05 or ^##^*p* < 0.01 compared with control group and ^*∗*^*p* < 0.05 or ^*∗∗*^*p* < 0.01 compared with RS model group. One-way ANOVA with Student–Newman–Keuls *post hoc* test.

The one-way ANOVA indicated significant differences among groups in the number of positive cells of CB1 (*F* (5, 42) = 6.740, *p* < 0.01) and CB2 (*F* (5, 42) = 16.901, *p* < 0.01) in the hippocampus CA1 region of rats. As shown in Figure 4, SWHP at doses of 1.5 g/kg and 3.0 g/kg significantly increased the number of positive cells of CB1 (*p* < 0.01, *p* < 0.01) and CB2 (*p* < 0.01, *p* < 0.05) compared with the RS group. Diazepam (1.0 mg/kg) significantly increased the number of positive cells in CB1 and CB2 compared with the control group (*p* < 0.01, *p* < 0.01).

### 3.4. Effects of SWHP on the BDNF Expression in RS Rats

**Figure 5:**
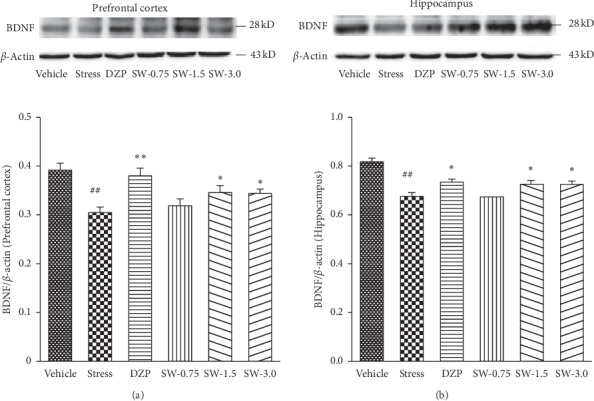
Effects of SWHP on BDNF expression of restraint stress rats in hippocampus and prefrontal cortex. Data represent mean ± SEM. ^#^*p* < 0.05 or ^##^*p* < 0.01 compared with control group and ^*∗*^*p* < 0.05 or ^*∗∗*^*p* < 0.01 compared with RS model group. One-way ANOVA with Student–Newman–Keuls *post hoc* test.

The one-way ANOVA indicated significant differences of BNDF expression in the prefrontal cortex (*F* (5, 42) = 7.024, *p* < 0.01) and hippocampus (*F* (5, 42) = 14.710, *p* < 0.01) among groups. As shown in Figure 5, BDNF expression was increased in the prefrontal cortex and hippocampus in the SWHP 1.5 g/kg and 3.0 g/kg dose groups when compared with the RS group (all *p* < 0.05). Similar effects were observed when treated with diazepam at a dose of 1.0 mg/kg (*p* < 0.01 in the prefrontal cortex; *p* < 0.05 in the hippocampus). The results showed that RS decreased the expression of BNDF. These effects were reversed by treatment with SWHP at doses of 1.5 g/kg and 3.0 g/kg. Therefore, SWHP may protect the prefrontal cortex and hippocampus from anxiety rats by promoting the expression of BDNF, resulting in corresponding antianxiety effects.

### 3.5. Effects of SWHP on the ERK, Phosphorylation of ERK, CREB, and Phosphorylation of CREB Expression in RS Rats

**Figure 6:**
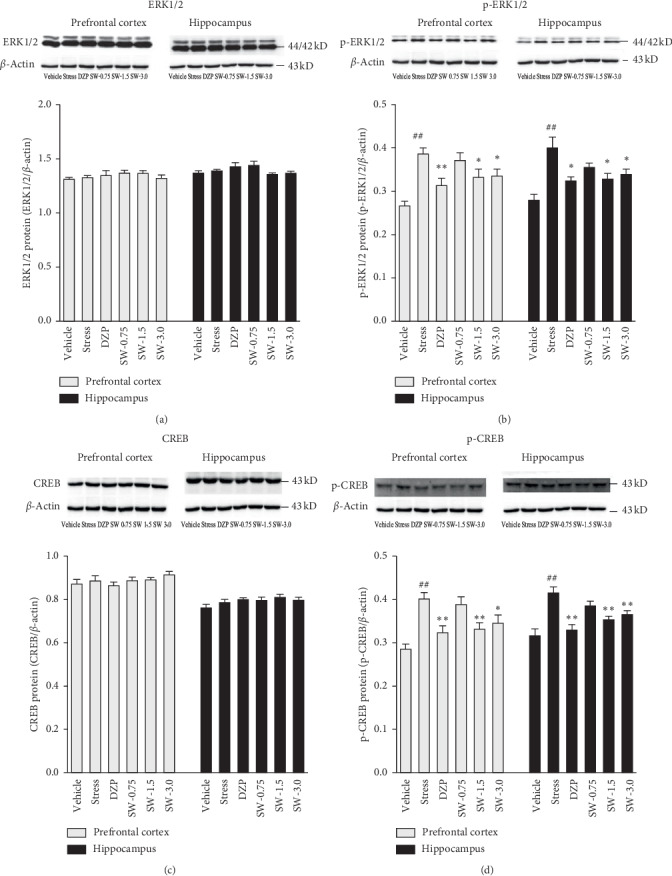
Effects of SWHP in restraint stress rats on ERK1/2, p-ERK1/2, CREB, and p-CREB expression. Data represent mean ± SEM. ^#^*p* < 0.05 or ^##^*p* < 0.01 compared with control group and ^*∗*^*p* < 0.05 or ^*∗∗*^*p* < 0.01 compared with restraint stress model group. One-way ANOVA with Student–Newman–Keuls *post hoc* test.

As shown in Figure 6, the one-way ANOVA revealed significant differences between p-ERK and p-CREB expression in prefrontal cortex (*F* (5, 42) = 6.945, *p* < 0.01; *F* (5, 42) = 7.440, *p* < 0.01) and hippocampus (*F* (5, 42) = 7.323, *p* < 0.01; *F* (5, 42) = 8.930, *p* < 0.01) among groups. RS significantly increased phosphorylation of ERK (p-ERK) in the prefrontal cortex and hippocampus when compared to control groups (both *p* < 0.01). The increased p-ERK was able to be reversed by administration of SWHP at doses of 1.5 g/kg and 3.0 g/kg in the prefrontal cortex and hippocampus (all *p* < 0.05).

Western blot analyses showed that phosphorylation of CREB (p-CREB) was significantly increased in the prefrontal cortex and hippocampus after RS when compared with control groups (both *p* < 0.01). The p-CREB expression in these two brain regions was reversed by treatment with SWHP at doses of 1.5 g/kg and 3.0 g/kg (in prefrontal cortex, *p* < 0.01 and *p* < 0.05; in hippocampus, both *p* < 0.01).

## 4. Discussion

Herb-pairs are the basic composition units of Chinese herbal formulae, usually consisting of two TCM herbs [13]. The herb-pairs, which are a unique clinical combination of two relatively fixed herbs, are the simplest and most fundamental form of multiherb therapy aimed at specific illnesses [37]. After being prescribed in combination, herb-pairs are used either to obtain synergistic effects or to diminish possible adverse reactions. The TCM formula does not mean a simple quantitative addition of different herbs, but reasonable and necessary interactions between herbs with specific functions [38, 39]. So, this also follows the “seven relations of TCM” theory, in which there are several aims and principles of herbal compatibility. These include singular application, mutual promotion, mutual assistance, mutual restraint, mutual detoxification, mutual inhibition, and mutual intoxication [40]. The principle of mutual promotion explains why herb-pairs have significantly better pharmacological efficacy than individual herbs and why these always demonstrate better curative effects than when used alone [13, 41].

As we know, herb-pairs are simpler in composition than complete formulae but are still therapeutically effective [37], which is an advantage of using herb-pairs. Studies found that Suanzaoren and Wuweizi are usually combined with a variety of Chinese medicine herbs in clinics for use as sedatives and tranquilizers. More than 17.8% Chinese medicine formulae of treating insomnia recorded in *Pharmacopeia of the People's Republic of China* use SWHP [42]. Many classical traditional formulae for insomnia like Tianwang Buxin Dan, Zhusha Anshen Wan, Baizi Yangxin Pills, and Suanzaoren Decoction would contain SWHP. Some studies have shown that this herb-pair has definite sedative and hypnotic effect on compatible application and also revealed related mechanisms of actions of these herbs [17–20]. As our group previously researched, by designing the orthogonal table and using the extract method, the compatibility and dosage as an examining index was in accordance to previous literature. The pharmacodynamics of the mice was understood according to orthogonal table. The results showed that SWHP has better antianxiety effect when extracted by alcohol-water and the compatibility proportion was 2 : 1. The dosage should be set to 0.75 g/kg, 1.5 g/kg, and 3 g/kg for subsequent research on the basis of the screening results. Although the pharmacological efficacy and curative effects of herb-pairs are better than single herb, we unfortunately have not further verified the antianxiety effect of Suanzaoren or Wuweizi when used alone compared with SWHP. We plan to conduct following research to explore whether SWHP has a better antianxiety effect than Suanzaoren or Wuweizi when used alone. A total of 30 compounds were identified in our early study, and we plan to explore which active ingredients of SWHP may be responsible for the antianxiety effect for further research. In this study, we only found that the ECS-BDNF-ERK signaling pathway could be activated. However, there is more participation of other receptors; e.g., the 5-HT1A receptor is also a target of anxiety. So, we would explore the mechanism in depth.

Animal stress models are the most common form of anxiety animal models. Stress models could simulate different physiological and emotional responses of stress in humans. RS is a nontraumatic stimulus model, which is similar to psychosomatic disease in humans. RS induces anxiety reactions such as fright, increased defecation, frequent modification, and erected hair in animals. The study showed that p-ERK1/2 levels in the prefrontal cortex were altered by acute and repeated restraint stress [43]. This could better simulate the behavior of human anxiety. So far, RS has proved to be an effective anxiety model; thus the RS model is one of the most commonly used [44, 45]. In this paper, we used RS-induced anxiety disorder model, which is well established. We observed that RS can reduce the time spent and the number of central entries in the OF test (Figure 2) and decrease the percentage of entries in open arms and time spent in open arms in EPM test (Figure 3). This model was used to analyze the anxiolytic-like effects and related mechanisms of SWHP.

The imbalance of ECS is related to a variety of CNS and immune system diseases. Two G-protein-coupled receptors (termed as CB1 and CB2) have been discovered to bind endocannabinoids and exogenous cannabinoid ligands [46]. CB1 and CB2 both had contact with many signal pathways [47, 48]. It was found that CB1 and CB2 receptors were highly expressed in the hippocampus. The regions with the highest expression of CB1 receptors were CA1 and CA3, and the regions with the highest expression of CB2 receptors were CA1 and CA2 [49]. In the hippocampus, enhanced ECS function may also produce anxiety relief and antidepressant effects, which are related to the hypothalamic-pituitary-adrenal axis function. Studies have found that cannabinoid substances bind to cannabinoid receptors, which activate signaling pathways in multiple cells, including MAPK/ERK pathways. The present study showed that RS could induce the decline of the ECS system functions (Figure 4), resulting in the number of CB1 and CB2 positive cells in the hippocampal CA1 region significantly decreasing (Figure 4). SWHP could increase the expression of CB1 and CB2, thereby producing antianxiety effect. So, SWHP may play an antianxiety role through the endocannabinoid system and those pathways signals, like the MAPK/ERK pathway, may be activated.

BDNF is one of the most widely studied neurotrophic factors in anxiety disorders and is related with neuroplasticity cellular mechanism. MAPK and PI3K are the two main signaling pathways of BDNF, with the MAPK signaling pathway being more active. BDNF has been shown to play a role in antidepressant treatments in major depressive disorder (MDD) and also has an effect in generalized anxiety disorder (GAD) [24]. A review paper showed that stress can have potent effects on the expression of neurotrophins, in particular, BDNF [50]. In clinical and animal models, BDNF has been associated with the development of affective disorders including MDD, anxiety disorders, and posttraumatic stress disorders. BDNF is closely related to the pathophysiology of anxiety disorders and has been used as a potential target for many antianxiety drugs [51–54]. A study found that BDNF is mainly synthesized by brain tissue and primarily distributed in the hippocampus and cortex in the CNS. The CNS regulates the plasticity of neurons and synapses and is of great significance for the proliferation and repair of central neurons. This is particularly important as the treatment of anxiety disorders is also closely related to the formation of neurons and synapses. In our study, the expression of BDNF in the brain tissue was detected by western blot. The results showed that SWHP could significantly counteract the onset of BDNF expression decrease (Figure 5). Therefore, SWHP could protect the prefrontal cortex and hippocampus in the anxiety rats by promoting the expression of BDNF, resulting in corresponding antianxiety effects.

ERK is a member of the MAPK family. The ERK signaling pathway is recognized as the classical MAPK signaling pathway. It is specifically associated with 5-HT1A receptors and is usually activated by growth factor tyrosine kinase receptors [55]. By this cascade reaction, biological effects are created: RAs activated ⟶ Rafl activated ⟶ MEK activated ⟶ ERK1/2 activated ⟶ RSK activated ⟶p-CREB ⟶ creates biological effects. The results of one study showed that physical and emotional stress could induce the p-ERK1/2 upregulation in central neurons. ERK1/2 is an important signaling molecule in the CNS, when there is stress-induced activity in the brain. P-ERK1/2 in its active form is a new marker used to indicate neuronal activation in functional morphology studies [25]. The cAMP-response element binding protein (CREB) is one of the important protein factors regulating the CNS and is widely distributed in the hippocampus and cortex. As we know, the ERK-CERB signaling pathway is thought to be associated with anxiety [56]. A study indicated that the levels of p-ERK were significantly increased during anxiety, and the results suggested that the ERK signal transduction pathway might play an important role in anxiety, suggesting that the inhibition of the ERK pathway phosphorylation could produce an anxiolysis effect [57]. The results of our study indicate that RS model activated ERK-CREB signaling pathway, which increased the p-ERKl/2 and p-CREB (Figure 6), and SWHP significantly inhibited the hyperphosphorylation of ERK1/2 and CREB (Figure 6). So, the effects of SWHP on anxiety-related behaviors might be mediated by the ERK-CREB signaling pathway. Therefore, the ECS influences on the MAPK/ERK signaling pathways, as well as BDNF and p-CREB, are relevant to anxiety-like behaviors and are downstream effector proteins [58].

In summary, this present study demonstrated that SWHP treatment may target the mechanism of the ECS-BDNF-ERK signaling pathway, which has an antianxiety effect in RS rat models. The results showed that SWHP could improve anxiety-like behaviors of RS-induced anxiety disorders in rat models, which was shown in the behavior tests. There was an increase in the expression of CB1 and CB2, which promoted the expression of BDNF and inhibited the hyperphosphorylation of ERK1/2 and CREB. The different dosages did not significantly affect the activity. So, the antianxiety effect mechanism of SWHP is generated through regulation of ECS-BDNF-ERK signaling pathway expression. Herb-pairs are characterized as multicomponent, multitarget, and multichannel. SWHP may affect anxiety disorder through a variety of active ingredients and pathways, so in our following research we will study the SWHP in other anxiety disorder related pathways as well as the antianxiety chemical constitutions of SWHP. Overall, the current data suggests that SWHP may be a candidate for developing therapeutic strategies for the anxiety disorder.

## 5. Conclusions

Our study demonstrated that SWHP improved anxiety-like behaviors in RS rat models by regulating the ECS-BDNF-ERK signaling pathway. The antianxiety effect of SWHP on rats with RS was evaluated by using the OF and EPM tests. The findings indicate that SWHP may have a therapeutic application in the RS model of anxiety disorder, which proposes a potential new direction for research into anxiety disorders regarding mechanisms and the development of novel antianxiety drugs.

This study demonstrated that Suanzaoren-Wuweizi herb-pair treatment may target the mechanism of the ECS-BDNF-ERK signaling pathway, which has an antianxiety effect in RS rat models. The antianxiety effect mechanism of Suanzaoren-Wuweizi herb-pair is produced through regulation of ECS-BDNF-ERK signaling pathway expression. Overall, the current data suggests that SWHP may be a candidate for developing therapeutic strategies for the anxiety disorder.

## Figures and Tables

**Table 1:** UPLC-LTQ Orbitrap MS of SWHP.

No.	Ion mode	tR (min)	Molecular weight (*m*/*z*)	Molecular formula	Fragment ions (*m*/*z*)	Chemical compound
1	Negative	4.59	191.01906	C_6_H_8_O_7_	172,130,128,110,86	Citric acid/isocitrate
2	Negative	9.85	191.019	C_6_H_8_O_7_	172,130,128,110	Citric acid/isocitrate
3	Negative	10.4	205.03429	C_7_H_10_O_7_	173,143,131,111	6-Methyl citrate
4	Negative	14.35	219.04974	C_8_H_12_O_7_	173,157,143,131,111	1,5-Dimethylcitrate
5	Negative	34.93	342.16934	C_20_H_24_NO_4_	297,282,265,237	Zizyphusine
6	Negative	73.59	543.22198	C_29_H_36_O_10_	525,499,481,445	Lancifodilactone C
7	Positive	73.71	501.34177	C_28_H_36_O_8_	455,437	Tigloylgomisin H or aegeloygomisin H
8	Positive	81.64	531.25757	C_29_H_38_O_9_	495,453,425	Angeloygomisin Q
9	Positive	84.94	389.19424	C_22_H_29_O_6_	374,358,342,319	Gomisin J
10	Positive	87.41	515.22632	C_28_H_34_O_9_	469,385,355	Tigloylgomisin P
11	Negative	87.41	401.1593	C_22_H_26_O_7_	354,284,270,257,255,242	3′,4′-Dimethoxybenzoicacid-(3″,4″-dimethoxyphenyl)-methyl-3-oxobutyl ester
12	Positive	87.94	523.22839	C_30_H_34_O_8_	508,493,477,386,315	Benzoylgomisin H
13	Positive	88.36	391.21078	C_22_H_30_O_6_	359,327,289,237,235,205,166	Pregomisin
14	Positive	90.06	523.22894	C_30_H_34_O_8_	493,386,315	Benzoylgomisin H isomer
15	Positive	91.75	387.17938	C_22_H_26_O_6_	372,358,357,356,355,313	Gomisin L2
16	Positive	94.95	403.31021	C_23_H_30_O_6_	388,372,371,340,333,302,301	Schisanhenol
17	Positive	95.41	403.21021	C_23_H_30_O_6_	388,372,371,356,340,333,301	Gomisin K1
18	Negative	96.74	537.20831	C_30_H_34_O_9_	415,385,371	Gomisin G
19	Positive	97.99	403.21021	C_23_H_30_O_6_	388,372,371,356,340,333,301	Gomisin K2
20	Positive	98.66	515.22552	C_28_H_34_O_9_	469,385,355,343,323	Schisantherin B or schisantherin C
21	Positive	99.73	515.22595	C_28_H_34_O_9_	385,355,316	Gomisin E
22	Positive	100.21	387.17983	C_22_H_26_O_6_	355,325,317	Gomisin M1
23	Positive	101.04	387.17896	C_22_H_26_O_6_	355,325,317	Gomisin M2
24	Negative	102.99	485.32553	C_30_H_45_O_5_	439,423	Ceanothic acid
25	Positive	103.65	417.22552	C_24_H_32_O_6_	402,386,370,347,316	Schisandrin A
26	Positive	105.69	401.19507	C_23_H_28_O_6_	386,371,370,331,300	Schisandrin B
27	Positive	106.25	33.117	C_20_H_26_O_4_	300,299,286	Meso-dihydroguaiaretic acid
28	Positive	106.40	401.19485	C_23_H_28_O_6_	386,371,370,331,300	Schisandrin B
29	Positive	107.65	385.16333	C_22_H_24_O_6_	370,355,315,284	Schisandrin C
30	Negative	113.6	279.2319	C_18_H_32_O_2_	261,259,243,83	9,12-Linoleic acid

## Data Availability

The data used to support the findings of this study are available from the corresponding author upon request.
